# Association between stress hyperglycemia ratio and prognosis in acute ischemic stroke: a systematic review and meta-analysis

**DOI:** 10.1186/s12883-023-03519-6

**Published:** 2024-01-02

**Authors:** Zhuoya Jiang, Kunyu Wang, Hanying Duan, Heqian Du, Su Gao, Jing Chen, Shaokuan Fang

**Affiliations:** Department of Neurology, Neuroscience Centre, the First Hospital of Jilin University, Changchun, China

**Keywords:** Acute ischemic stroke, Stress hyperglycemia ratio, Stress hyperglycemia, HbA1c, Meta-analysis

## Abstract

**Background:**

Stress hyperglycemia is a relatively transient increase in blood glucose in response to inflammation of the body and neurohormonal disorders. It is still debated whether stress hyperglycemia ratio (SHR) in the acute phase, a new indicator of stress hyperglycemia, is related to poor prognosis in acute ischemic stroke (AIS) patients. This meta-analysis provides insight into the connection between SHR and prognosis in AIS patients.

**Methods:**

We screened all potentially relevant studies using a comprehensive database search. The standardized mean difference (SMD) and 95% confidence interval (CI) were utilized to investigate the relationship between SHR in the acute phase and the prognosis of AIS.

**Results:**

The pooled results revealed that AIS patients with poor prognoses had significantly higher SHR values than those with good prognoses (SMD = 0.56, 95%CI: 0.37–0.75, *p*<0.001). Subgroup analysis indicated that study design and differences in post-stroke treatment might be the sources of heterogeneity in this meta-analysis.

**Conclusions:**

High SHR in the acute period is related to poor prognosis after AIS. SHR may be a new predictor of poor outcomes in AIS patients.

**Supplementary Information:**

The online version contains supplementary material available at 10.1186/s12883-023-03519-6.

## Introduction

Globally, stroke is a significant cause of death and disability [1, 2]. The Global Burden of Diseases, injury, and Risk Factors Study (GBD) showed a substantial increase in stroke events attributable to exposure to risk factors from 1999 to 2019. In 2019, the number of people who died from stroke accounted for 11.6% of all deaths, and stroke remains the second leading cause of death and the third leading cause of death and disability combined [3]. In addition, a large sample survey showed that in 2020, the estimated prevalence of stroke in people aged ≥ 40 years in China was 2.6%, the incidence rate was 505.2/100 000 person-years, and the mortality rate was 343.4/100 000 person-years [4].

Hyperglycemia is a risk factor for stroke [5]. Stress hyperglycemia is a relatively transient increase in blood glucose in response to inflammation of the body and neurohormonal disorder [6, 7]. As a result of the sympathetic nervous system and the hypothalamic-pituitary-adrenal axis being engaged during a stroke, catecholamines and cortisol are released [7, 8]. Stress hormone disorders inhibit insulin secretion and promote glycogenolysis and hepatic gluconeogenesis, resulting in a relatively transient increase in blood glucose level [6, 9]. Absolute hyperglycemia does not distinguish between chronic poor glycemic management and stress response in acute ischemic stroke(AIS). Roberts et al. proposed the concept of relative hyperglycemia - stress hyperglycemia ratio (SHR) [10]. Compared to absolute hyperglycemia, stress hyperglycemia ratio is a more predictive prognostic marker. Studies have demonstrated a significant correlation between elevated stress hyperglycemia ratio, measured as fasting blood glucose/glycosylated hemoglobin (FBG/HbA1c), and the poor outcomes of AIS patients [11, 12], including an increased risk of functional impairments, stroke recurrence, and death [13–15]. However, Nathan et al. suggested using fasting blood glucose/estimated average glucose (FBG/EAG) to define the stress hyperglycemia ratio, where EAG= (1.59 ×HbA1c) -2.59 [16]. Two previous studies have shown that SHR calculated as FBG/EAG predicts poor outcomes in patients with AIS or critical illness [17, 18], suggesting that elevated SHR may predict poor clinical prognosis in AIS patients.

However, there are few studies on the association between SHR and prognosis in patients with AIS, and the underlying pathological mechanisms have not been thoroughly investigated. Still, there are several possible explanations: (1) Hyperglycemia during cerebral ischemia affects the energy metabolism of brain cells and aggravates anaerobic glycolysis, which can lead to intracellular acidosis [6]. Intracellular acidosis can further exacerbate ischemic brain injury [7]; (2) At the time of ischemic stroke, glutamate accumulates extracellular and activates postsynaptic glutamate receptors in a hyperglycemic state [10]. The excessive opening of Ca2 + channels leads to intracellular Ca2 + overload and impaired mitochondrial function, which eventually leads to neuronal death [6]; (3) After ischemic stroke, hyperglycemia promotes inflammatory response and oxidative stress and activates matrix metalloproteinase-9 activity [9]. This series of reactions further disrupts the blood-brain barrier, exacerbates cerebral edema, and causes hemorrhagic transition [19, 20]. (4) Elevated blood glucose leads to vascular endothelial dysfunction in patients with AIS [11], and the decrease of cerebral blood flow further aggravates ischemic injury [19]. (5) Hyperglycemia may directly damage the ischemic penumbra by transferring intracellular electrolytes [21, 22]. This cascade of reactions can form a vicious cycle affecting stroke patients’ short-term and long-term neurological recovery. Indeed, many factors complicate the prognosis of ischemic stroke, and their relevance requires further investigation. In light of this, we performed the meta-analysis to investigate the significance of acute phase SHR in predicting clinical outcomes in AIS patients.

## Methods

The meta-analysis was carried out using the Preferred Reporting Items for Systematic Reviews and Meta-analyses guidelines (PRISMA) [23].

### Search strategy

We searched eight databases, including PubMed, Cochrane Library, Embase, Wed of Science, Chinese National Knowledge Infrastructure (CNKI), China Biology Medicine Literature Database (CBM), Wan fang, and VIP Chinese Journal Database, until October 2023. Publications are limited to Chinese and English. The search strategies were as follows: (“ischemic stroke” OR “acute ischemic stroke”) AND (“stress hyperglycemia” OR “stress hyperglycemia ratio” OR “stress hyperglycaemia” OR “stress hyperglycaemia ratio” OR “hyperglycemia” OR “hyperglycaemia” OR “glycated hemoglobin A” OR “HbA1C” OR “glycated hemoglobin” OR “glycosylated hemoglobin”). Finally, references to selected articles were browsed for potentially relevant research. The detailed search strategy were presented in Supplementary material.

### Selection criteria

Inclusion criteria: (1) Cohort study; (2) Patients in the study were diagnosed with AIS; (3) Experiments and controls were designed to compare SHR in the good and poor prognosis groups; (4) SHR was calculated as fasting blood glucose (mmol/L)/HbA1c (%) (FBG/HbA1c) or fasting blood glucose (mmol/L)/estimated average glucose (FBG/EAG); (5) Prognostic evaluation of patients with AIS used modified Rankin scale (mRS) [24, 25]; (6) Research data were available. Exclusion Criteria: (1) Duplicate studies; (2) Review, meta-analysis, animal experiment, case report, letter, and conference abstract were excluded. When the study populations overlapped in multiple publications, we included the most recent or complete one. Two researchers independently completed the literature screening and reached a consensus through discussion.

### Data extraction

The following data were extracted independently by two researchers: first author, publication year, study design, country, the proportion of men, diabetes status, post-stroke treatment, the time point of outcome assessment, prognostic outcome, and definition of SHR. When SHR was expressed as the median and interquartile range (IQR), SHR was transformed to mean and standard deviation (SD) using the quantile estimation (QE) method of Sean McGrath et al. [26]. Any disagreements were resolved through group discussions.

### Quality assessment

The literature’s quality was assessed using the three criteria of selection, comparability, and outcome using the Newcastle-Ottawa Quality Assessment Scale (NOS) [27]. Each star represents 1 point, and a score of 5 or more is considered high quality. We awarded up to 1 star per category for “selection” and “outcome” and up to 2 stars for “comparability”. Discussions resolved discrepancies in quality assessment among investigators.

### Statistical analysis

STATA 16.0 was used to analyze the data. Forest plots of continuous data were constructed using SMD and 95% CI. Heterogeneity was examined using the I^2^ statistic. The heterogeneity was considered significant when I square(I^2^) > 50%. If significant heterogeneity is detected, random-effects models should be used. We conducted a sensitivity analysis when there was significant inter-study heterogeneity to see how each study’s results might affect the overall effect size. Subgroup analysis and meta-regression analysis were employed to investigate potential sources of heterogeneity.

Additionally, we also performed funnel plots and Egger’s test to assess publication bias.

## Results

### Literature research

Supplementary Fig. 1 shows a flow chart of study search and screening. Eight electronic databases were searched, and 2 844 articles were obtained. First, 359 copies were eliminated. After carefully reading the titles and abstracts, 2,459 pieces were removed, including some animal studies, reviews, reviews, correspondence, case reports, meta-analyses, and articles not relevant to this study. 16 of the 26 remaining papers were removed after evaluation because they didn’t meet the criteria for inclusion. Ten articles were ultimately included.

### Study characteristics and quality assessment

Supplementary Table 3 presents the main features of this study. Shen et al. evaluated SHR using both FBG/HbA1c and FBG/EAG [28]. Thus, ten articles were included, including 11 cohort studies investigating the relationship between SHR and AIS prognosis. Among them, one study was conducted in Singapore [29], one study was conducted in Australia [30], and the remaining studies were conducted in China [17, 28, 31–36]. For the evaluation of SHR, FBG/HbA1c was used in five studies [28, 29, 31–34] while FBG/EAG was used in the remaining five [28, 30, 33, 35, 36]. Eight studies performed intravenous thrombolysis or mechanical thrombectomy after a stroke [17, 28–30, 33–36], while the other two did not [31, 32]. Eight studies evaluated patient outcomes three months after stroke [17, 28, 29, 32–36], one study evaluated patient outcomes six months after stroke [31] and one study evaluated stroke outcomes at discharge [30]. The quality scores of the selected studies were acceptable, with NOS scores of 7–9 points (Supplementary Table 2).

### Relationship between SHR and the prognosis of AIS

To assess the connection between SHR and clinical prognosis in AIS patients, a total of 11 cohort studies were incorporated. The post-stroke prognosis was assessed by Modified Rankin Scale(mRS) [24, 25], including 3,390 patients with good outcomes and 2,000 patients with poor outcomes. The result showed that AIS patients with poor prognoses had significantly higher SHR values than those with good prognoses (SMD = 0.56, 95%CI: 0.37–0.75, *p*<0.001) (Supplementary Fig. 2). Due to significant heterogeneity among studies (I^2^ = 90.0%, *p*<0.001), SMDs were pooled based on a random effect model.

### Sensitivity analysis

After a particular study was eliminated sequentially, the corresponding SMD values neither reversed the result nor changed significantly (Supplementary Fig. 3). This indicates that the result of this meta-analysis is relatively stable.

### Subgroup analysis

Seven subgroup analyses (including study design, country, diabetes status, post-stroke treatment, the time point of outcome assessment, the definition of SHR, and presentation of primary data) were conducted to explore factors affecting heterogeneity according to clinical characteristics of patients and study methodology. Supplementary Table 4 presents the results of the subgroup analysis. The results of the seven subgroup analyses were statistically significant, and in the poor outcome group, compared to the good outcome group, the SHR value was considerably higher. Heterogeneity was reduced to varying degrees in most subgroup analyses, particularly in study design and poststroke treatment subgroups. Subgroup analysis showed that study design (RC: SMD = 0.50, 95%CI: 0.29–0.71, I^2^ = 86.8%; PC: SMD = 0.72, 95%CI: 0.53–0.92, I^2^ = 68.6%) and differences in post-stroke treatment (IVT or MT: SMD = 0.51, 95%CI: 0.33–0.70, I^2^ = 86.3%; none: SMD = 0.80, 95%CI: 0.57–1.04, I^2^ = 42.7%) might be the sources of heterogeneity.

### Meta-regression analysis

Meta-regression analysis showed that study design, country, diabetes mellitus, post-stroke treatment, the time point of outcome assessment, the definition of SHR, and original data presentation were not the source of heterogeneity (Supplementary Table 5).

### Publication bias

To evaluate publication bias, we created a funnel plot (Supplementary Fig. 4). Subjectively, the scatter in the funnel plot is less symmetric, with more scatter on the right side. The Egger’s test (Supplementary Fig. 5) confirmed no significant publication bias.

## Discussion

### SHR and the prognosis of AIS

Comprehensive searches of eight electronic databases led to the selection of ten articles, comprising eleven cohort studies investigating the connection between SHR and prognosis in AIS patients. Six articles have demonstrated that poor short-term outcomes following AIS were associated with higher SHR in the acute period [17, 28, 29, 32, 33, 35]. Two articles suggested that SHR could independently predict short-term poor outcomes [28, 32]. In addition, one piece suggested a correlation between SHR and poor long-term outcomes in AIS patients [31]. Our findings showed that high SHR in the acute phase might predict poor outcomes after AIS. The finding was demonstrated to be reliable and stable by sensitivity analysis and publication bias analysis.

### Discussion on the sources of heterogeneity

At the same time, significant heterogeneity is worth exploring. Meta-regression analysis showed that study design, country, diabetes mellitus(DM), post-stroke treatment, the time point of outcome assessment, the definition of SHR, and original data presentation were not the source of heterogeneity.

Based on clinical characteristics and study methodology, we continued to perform subgroup analyses of the included studies into seven categories. The results were statistically significant in each subgroup, with the SHR value being considerably higher in the poor outcome group than in the good outcome group. Heterogeneity was reduced to varying degrees in most subgroup analyses, particularly in study design and poststroke treatment subgroups.

Differences in study design may be one source of heterogeneity. Of the eleven studies, eight were retrospective cohort studies, and the remaining three were prospective cohort studies. Subgroup analysis indicated that the reduction in heterogeneity was more pronounced in the prospective study compared to the retrospective study. The main reason is that retrospective studies are more biased, while prospective studies are more scientific.

Different poststroke interventions may be another source of heterogeneity. In nine of the eleven studies, intravenous thrombolysis (IVT) or mechanical thrombectomy (MT) was performed after the stroke, while the remaining two did not. The subgroup that did not use IVT or MT showed a significant reduction in heterogeneity. Currently, the main treatments for acute ischemic stroke are IVT, MT, and antiplatelet therapy. In clinical practice, however, there are still patients with varying degrees of poor prognosis, even if the corresponding treatment is given proactively. According to previous studies, elevated SHR in the acute period is linked to a poorer functional outcome in AIS patients after IVT or MT [12, 17, 29], as well as increased risk of stroke recurrence [14] and post-stroke hemorrhagic transition [10, 37]. Stress hyperglycemia promotes oxidative stress and inflammatory response, reducing collateral circulation in the ischemic penumbra surrounding the infarction and transforming the ischemic penumbra into irreversible infarction [8, 38, 39]. Even if the vessel is realized in time, it may result in a poor clinical prognosis [37]. However, the conclusions of these studies are limited. More research is still required to fully understand the connection between SHR and prognosis in patients with AIS, given that mechanical thrombectomy and intravenous thrombolysis may somewhat affect patients’ prognosis trajectories.

When stratified by the proportion of diabetes in the study population, the heterogeneity in the subgroup with < 30% diabetes was significantly reduced. This suggests that the findings of this meta-analysis may be more relevant in people without diabetes. Absolute hyperglycemia does not reflect glycemic stress changes in critical situations. The relative stress hyperglycemia index-SHR considers background glucose and is a good predictor of poor outcomes in acute patients [28]. Previous research has revealed that the likelihood of recurrence in people with mild stroke or transient ischemic attack is the same for newly diagnosed and previously diagnosed diabetes [40]. In contrast, in patients with stress hyperglycemia, the risk is significantly higher [40]. Merlino et al. showed that SHR was related to poor outcomes after IVT in AIS patients, regardless of diabetes [12]. However, Zhang et al. verified that SHR is an independent predictor of poor outcomes following AIS and that the predictive effect is particularly notable in non-diabetic individuals [35]. In addition, it has been revealed that stress hyperglycemia only leads to adverse outcomes in non-diabetic patients, as people with diabetes chronically exposed to high glucose levels may have better cellular adaptation and response to hyperglycemia [41]. Therefore, the predictive value of SHR in AIS patients with or without diabetes remains to be investigated further.

Heterogeneity was significantly reduced when FBG/HbA1c was used to assess SHR. Previous studies used receiver operating characteristic(ROC) curves to determine the predictive value of admission blood glucose, FBG, HbA1c, FBG/HbA1c, and FBG/EAG for poor clinical prognosis three months after AIS. The results showed that the stress hyperglycemia ratio as measured by FBG/HbA1c and FBG/EAG was independently related to poor outcomes in AIS patients and that FBG/HbA1c was a better predictor of poor prognosis in AIS patients than blood glucose, FBG, HbA1c and FBG/EAG at admission [28]. Therefore, FBG/HbA1c may be a more appropriate prognostic predictor of AIS than FBG/EAG.

Due to the need for meta-analysis, the median and interquartile range of the raw data had to be converted to mean and standard deviation. Therefore, this heterogeneity is inevitable.

### Clinical significance

Acute ischemic stroke is a common cerebrovascular disease with high incidence, disability, and mortality. Disability due to stroke imposes a considerable burden on individuals, families, and society, and early identification and management of high-risk patients are essential. Diabetes mellitus is a recognized risk factor for cerebrovascular disease. However, the consequences of stress hyperglycemia have not been fully established, and the association of stress hyperglycemia with poor functional outcomes is controversial. The result of this meta-analysis may shed some light on glycemic management in patients with AIS. Whether SHR can be a new predictor and target for early intervention still deserves further research.

### Limitations and prospects

First, the high degree of heterogeneity may reduce the reliability of the findings. Meanwhile, additional research is required to explore the association between SHR and the prognosis of AIS patients due to the dearth of included studies. Most of the studies included in this study were limited to China, and more studies from other regions are needed to verify the reliability and generalization of the conclusions of this study. Furthermore, just one of the studies we included had participants who were followed for six months, necessitating further research into how SHR affects stroke outcomes in the short and long term. Whether the association between SHR and stroke outcome is more pronounced in non-diabetic patients and whether FBG/HbA1c is a more appropriate predictor of AIS outcome compared to FBG/EAG remains to be investigated.

## Conclusion

In conclusion, the findings of this meta-analysis provided new insight into the connection between SHR and clinical outcomes in AIS patients. We found that elevated SHR in the acute phase was associated with a worse outcome, indicating that elevated SHR may predict poor clinical outcomes after AIS. Nonetheless, given the low number of included studies, more studies may be needed to explore the predictive value of SHR in AIS.

### Electronic supplementary material

Below is the link to the electronic supplementary material.

Supplementary Material 1

## Data Availability

All data are available and taken from the previously published article.

## Abbreviations

SHR: Stress hyperglycemia ratio
AIS: Acute ischemic stroke
SMD: Standardized mean difference
CI: Confidence interval
HbA1C: Glycosylated hemoglobin
FBG: Fasting blood glucose
EAG: Estimated average glucose
PRISMA: Preferred Reporting Items for Systematic Reviews and Meta-analyses
mRS: Modified Rankin Scale
IQR: Interquartile range
SD: Standard deviation
QE: Quantile estimation
NOS: Newcastle-Ottawa Quality Assessment Scale
I^2^: I square
RC: Retrospective cohort
PC: Prospective cohort
IVT: Intravenous thrombolysis
MT: Mechanical thrombectomy
DM: Diabetes mellitus
ROC: Receiver Operating Characteristic
CNKI: Chinese National Knowledge Infrastructure
CBM: China Biology Medicine Literature Database
