# Supplementary material for: Association between stress hyperglycemia ratio and prognosis in acute ischemic stroke: a systematic review and meta-analysis
Source: BMC Neurol. 2024 Jan 2;24:13. doi: 10.1186/s12883-023-03519-6 (PMC10759321; doi:10.1186/s12883-023-03519-6)

**Supplementary appendix**

Appendix Search strategy

Table S1. PRISMA 2020 checklist

Table S2. Quality assessment

Table S3. Characteristics of studies included

Table S4. Results of subgroup analyses

Table S5. Results of meta-regression analysis

Figure S1. Flowchart of study search and selection

Figure S2. Forest plot of the relationship between SHR and the prognosis of AIS

Figure S3. Sensitivity analysis of the meta-analysis

Figure S4. The funnel plot of the meta-analysis

Figure S5. Egger’s test of the meta-analysis

Search strategy

Search strategy for China Biology Medicine literature database (CBM)：

("ischemic stroke"[Commonly used field: Intelligence] OR "acute ischemic stroke"[Commonly used field: Intelligence]) AND ("stress hyperglycemia"[Commonly used field: Intelligence] OR "stress hyperglycemia ratio"[Commonly used field: Intelligence] OR "stress hyperglycaemia"[Commonly used field: Intelligence] OR "stress hyperglycaemia ratio"[Commonly used field: Intelligence] OR "hyperglycemia"[Commonly used field: Intelligence] OR "hyperglycaemia"[Commonly used field: Intelligence] OR "glycated hemoglobin A"[Commonly used field: Intelligence] OR "HbA1C"[Commonly used field: Intelligence] OR "glycated hemoglobin"[Commonly used field: Intelligence] OR “glycosylated hemoglobin” [Commonly used field: Intelligence]).

The retrieval strategies of CNKI (Chinese national knowledge infrastructure), Wan fang, and VIP Chinese Journal Database are modified on the basis of CBM retrieval strategy according to the characteristics of each database.

Search strategy for PubMed：

("ischemic stroke"[Title/Abstract] OR "acute ischemic stroke"[Title/Abstract]) AND ("stress hyperglycemia"[Title/Abstract] OR "stress hyperglycemia ratio"[Title/Abstract] OR "stress hyperglycaemia"[Title/Abstract] OR "stress hyperglycaemia ratio"[Title/Abstract] OR "hyperglycemia"[Title/Abstract] OR "hyperglycaemia"[Title/Abstract] OR "glycated hemoglobin A"[Title/Abstract] OR "HbA1C"[Title/Abstract] OR "glycated hemoglobin"[Title/Abstract] OR “glycosylated hemoglobin” [Title/Abstract]).

The retrieval strategies of EMBASE and Cochrane Library are modified on the basis of PubMed retrieval strategy according to the characteristics of each database.

Table S1. PRISMA 2020 checklist

| **1Section and Topic** | **Item #** | **Checklist item** | **Location where item is reported** |
| --- | --- | --- | --- |
| **TITLE** | | |  |
| Title | 1 | Identify the report as a systematic review. | 1 |
| **ABSTRACT** | | |  |
| Abstract | 2 | See the PRISMA 2020 for Abstracts checklist. | 2 |
| **INTRODUCTION** | | |  |
| Rationale | 3 | Describe the rationale for the review in the context of existing knowledge. | 3-4 |
| Objectives | 4 | Provide an explicit statement of the objective(s) or question(s) the review addresses. | 5 |
| **METHODS** | | |  |
| Eligibility criteria | 5 | Specify the inclusion and exclusion criteria for the review and how studies were grouped for the syntheses. | 6 |
| Information sources | 6 | Specify all databases, registers, websites, organisations, reference lists and other sources searched or consulted to identify studies. Specify the date when each source was last searched or consulted. | 5-6 |
| Search strategy | 7 | Present the full search strategies for all databases, registers and websites, including any filters and limits used. | 6 |
| Selection process | 8 | Specify the methods used to decide whether a study met the inclusion criteria of the review, including how many reviewers screened each record and each report retrieved, whether they worked independently, and if applicable, details of automation tools used in the process. | 6-7 |
| Data collection process | 9 | Specify the methods used to collect data from reports, including how many reviewers collected data from each report, whether they worked independently, any processes for obtaining or confirming data from study investigators, and if applicable, details of automation tools used in the process. | 7 |
| Data items | 10a | List and define all outcomes for which data were sought. Specify whether all results that were compatible with each outcome domain in each study were sought (e.g. for all measures, time points, analyses), and if not, the methods used to decide which results to collect. | 7 |
|  | 10b | List and define all other variables for which data were sought (e.g. participant and intervention characteristics, funding sources). Describe any assumptions made about any missing or unclear information. | 7 |
| Study risk of bias assessment | 11 | Specify the methods used to assess risk of bias in the included studies, including details of the tool(s) used, how many reviewers assessed each study and whether they worked independently, and if applicable, details of automation tools used in the process. | 7 |
| Effect measures | 12 | Specify for each outcome the effect measure(s) (e.g. risk ratio, mean difference) used in the synthesis or presentation of results. | 7 |
| Synthesis methods | 13a | Describe the processes used to decide which studies were eligible for each synthesis (e.g. tabulating the study intervention characteristics and comparing against the planned groups for each synthesis (item #5)). | 7-8 |
|  | 13b | Describe any methods required to prepare the data for presentation or synthesis, such as handling of missing summary statistics, or data conversions. | 7-8 |
|  | 13c | Describe any methods used to tabulate or visually display results of individual studies and syntheses. | 7-8 |
|  | 13d | Describe any methods used to synthesize results and provide a rationale for the choice(s). If meta-analysis was performed, describe the model(s), method(s) to identify the presence and extent of statistical heterogeneity, and software package(s) used. | 7-8 |
|  | 13e | Describe any methods used to explore possible causes of heterogeneity among study results (e.g. subgroup analysis, meta-regression). | 7 |
|  | 13f | Describe any sensitivity analyses conducted to assess robustness of the synthesized results. | 7 |
| Reporting bias assessment | 14 | Describe any methods used to assess risk of bias due to missing results in a synthesis (arising from reporting biases). | 7 |
| Certainty assessment | 15 | Describe any methods used to assess certainty (or confidence) in the body of evidence for an outcome. | 7-8 |
| **RESULTS** | | |  |
| Study selection | 16a | Describe the results of the search and selection process, from the number of records identified in the search to the number of studies included in the review, ideally using a flow diagram. | 8  Figure S1 |
|  | 16b | Cite studies that might appear to meet the inclusion criteria, but which were excluded, and explain why they were excluded. | 8  Figure S1 |
| Study characteristics | 17 | Cite each included study and present its characteristics. | 8-9  Table S3 |
| Risk of bias in studies | 18 | Present assessments of risk of bias for each included study. | 11  Table S2 |
| Results of individual studies | 19 | For all outcomes, present, for each study: (a) summary statistics for each group (where appropriate) and (b) an effect estimate and its precision (e.g. confidence/credible interval), ideally using structured tables or plots. | 9  FigureS2 |
| Results of syntheses | 20a | For each synthesis, briefly summarise the characteristics and risk of bias among contributing studies. | 11 |
|  | 20b | Present results of all statistical syntheses conducted. If meta-analysis was done, present for each the summary estimate and its precision (e.g. confidence/credible interval) and measures of statistical heterogeneity. If comparing groups, describe the direction of the effect. | 9 |
|  | 20c | Present results of all investigations of possible causes of heterogeneity among study results. | 10-11 |
|  | 20d | Present results of all sensitivity analyses conducted to assess the robustness of the synthesized results. | 10 |
| Reporting biases | 21 | Present assessments of risk of bias due to missing results (arising from reporting biases) for each synthesis assessed. | 11 |
| Certainty of evidence | 22 | Present assessments of certainty (or confidence) in the body of evidence for each outcome assessed. | 9-11 |
| **DISCUSSION** | | |  |
| Discussion | 23a | Provide a general interpretation of the results in the context of other evidence. | 11-15 |
|  | 23b | Discuss any limitations of the evidence included in the review. | 15-16 |
|  | 23c | Discuss any limitations of the review processes used. | 15-16 |
|  | 23d | Discuss implications of the results for practice, policy, and future research. | 15-16 |
| **OTHER INFORMATION** | | |  |
| 5Registration and protocol | 24a | Provide registration information for the review, including register name and registration number, or state that the review was not registered. | 17 |
|  | 24b | Indicate where the review protocol can be accessed, or state that a protocol was not prepared. | 17 |
|  | 24c | Describe and explain any amendments to information provided at registration or in the protocol. | 17 |
| Support | 25 | Describe sources of financial or non-financial support for the review, and the role of the funders or sponsors in the review. | 17 |
| Competing interests | 26 | Declare any competing interests of review authors. | 17 |
| Availability of data, code and other materials | 27 | Report which of the following are publicly available and where they can be found: template data collection forms; data extracted from included studies; data used for all analyses; analytic code; any other materials used in the review. | 17 |

*From:*  Page MJ, McKenzie JE, Bossuyt PM, Boutron I, Hoffmann TC, Mulrow CD, et al. The PRISMA 2020 statement: an updated guideline for reporting systematic reviews. BMJ 2021;372:n71. doi: 10.1136/bmj.n71

Table S2. Quality assessment based on the Newcastle–Ottawa Quality Assessment Scale (NOS) adapted for cohort studies

| **Reference** | **Selection** | | | | **Comparability** | **Outcome** | | | **Score** |
| --- | --- | --- | --- | --- | --- | --- | --- | --- | --- |
|  | Representativeness  of the exposed  cohort | Selection of the non-exposed cohort | Ascertainment of exposure | Demonstration that outcome of interest was not present at start  of study | Comparability of  cohorts on the basis  of the design or  analysis controlled  for confounders | Assessment of outcome | Was follow-up long enough  for outcomes  to occur | Adequacy of follow-up of cohort |  |
| Sun 2023 | ★ | ★ | ★ | ★ | ☆ | ★ | ★ | ★ | 7 |
| Li 2022 | ★ | ★ | ★ | ★ | ★★ | ★ | ★ | ★ | 9 |
| Zhang 2022 | ★ | ★ | ★ | ★ | ★★ | ★ | ★ | ★ | 9 |
| Shen 2022 | ★ | ★ | ★ | ★ | ★★ | ★ | ★ | ★ | 9 |
| Ngiam 2022 | ★ | ★ | ★ | ★ | ★★ | ★ | ★ | ★ | 9 |
| Chen 2022 | ★ | ★ | ★ | ★ | ★★ | ★ | ★ | ★ | 9 |
| Liu 2022 | ★ | ★ | ★ | ★ | ★★ | ★ | ★ | ★ | 9 |
| Roberts 2021 | ★ | ★ | ★ | ★ | ★★ | ★ | ☆ | ★ | 8 |
| Chen 2019 | ★ | ★ | ★ | ★ | ★★ | ★ | ★ | ★ | 9 |
| Xie 2018 | ★ | ★ | ★ | ★ | ★★ | ★ | ★ | ★ | 9 |

“★” represents 1 points , “☆” represents 0 points.

Table S3. Characteristics of studies included

| **Study** | **Design** | **Country** | **Male (%)** | **DM (%)** | **Treatment** | **Time points of**  **outcome assessment** | **Number** | | **SHR (mean ±SD)** | | **Definition of SHR** |
| --- | --- | --- | --- | --- | --- | --- | --- | --- | --- | --- | --- |
|  |  |  |  |  |  |  | **Good outcome**  **(mRS 0-2)** | **Pour outcome**  **(mRS 3-6)** | **Good outcome**  **(mRS 0-2)** | **Pour outcome**  **(mRS 3-6)** |  |
| Sun 2023 | RC | China | 59.1 | 13.9 | MT | 3 months after stroke | 231 | 192 | 0.83±0.16 | 0.98±0.22 | FBG/EAG |
| Li 2022 | RC | China | 62.4 | 32.6 | IVT | 3 months after stroke | 252 | 67 | 1.21±0.33 | 1.22±0.33 | FBG/HbA1c |
| Zhang 2022 | RC | China | 63.9 | 53.3 | IVT  MT | 3 months after stroke | 923 | 561 | 0.83±0.17 | 0.87±0.21 | FBG/EAG |
| Shen 2022 | PC | China | 70.7 | 22.6 | IVT | 3 months after stroke | 214 | 127 | 0.91±0.19 | 1.05±0.29 | FBG/HbA1c |
|  |  |  |  |  |  |  |  |  | 0.78±0.16 | 0.90±0.23 | FBG/EAG |
| Ngiam 2022 | RC | Singapore | 60.7 | 18.9 | IVT | 3 months after stroke | 361 | 305 | 0.88±0.20 | 0.99±0.26 | FBG/HbA1c |
| Chen 2022 | RC | China | 62.2 | 33.0 | IVT | 3 months after stroke | 158 | 72 | 0.88±0.18 | 1.04±0.26 | FBG/HbA1c |
| Liu 2022 | PC | China | 59.3 | 39.0 | None | 3 months after stroke | 812 | 474 | 0.87±0.14 | 1.03±0.24 | FBG/HbA1c |
| Roberts 2021 | RC | Australia | 53.0 | / | IVT | at discharge | 219 | 81 | 0.98±0.24 | 1.05±0.29 | FBG/EAG |
| Chen 2019 | RC | China | 67.5 | 18.1 | MT | 3 months after stroke | 71 | 89 | 0.85±0.19 | 1.03±0.20 | FBG/EAG |
| Xie 2018 | RC | China | 63.5 | 19.3 | None | 6 months after stroke | 149 | 32 | 0.93±0.15 | 1.03±0.23 | FBG/HbA1c |

Abbreviations: RC: retrospective cohort; PC: prospective cohort; DM: diabetes mellitus; IVT: intravenous thrombolysis; MT: mechanical thrombectomy; mRS: modified Rankin scale; SHR: stress hyperglycemia ratio; SD: standard deviation; FBG: fasting blood glucose; HbA1c:

Hemoglobin A1c; EAG: estimated average glucose; EAG=(1.59×HbA1c)-2.59; None: IVT or MT was not performed.

Table S4. Results of subgroup analyses

| Sample characteristic | No. of  studies | SMD (95% CI) | I^2^ value (%) | | P value for  heterogeneity | Z | P |
| --- | --- | --- | --- | --- | --- | --- | --- |
| Study design | | | | | | | |
| RC 8 0.50(0.29 to 0.71) 86.8 0.000 4.68 0.000 | | | | | | | |
| PC 3 0.72(0.53 to 0.92) 68.6 0.041 7.39 0.000 | | | | | | | |
| Country | | | | | | | |
| China 9 0.60(0.37 to 0.83) 91.6 0.000 5.14 0.000 | | | | | | | |
| non-China 2 0.40(0.21 to 0.60) 44.3 0.180 4.09 0.000 | | | | | | | |
| DM | | | | | | | |
| ≥30% 4 0.47(0.05 to 0.90) 96.3 0.000 2.18 0.029 | | | | | | | |
| ＜30% 6 0.65(0.52 to 0.78) 46.0 0.099 9.83 0.000 | | | | | | | |
| Poststroke treatment | | | | | | | |
| IVT or MT | 9 | 0.51(0.33 to 0.70) | | 86.3 | 0.000 | 5.47 | 0.000 |
| none | 2 | 0.80(0.57 to 1.04) | | 42.7 | 0.186 | 6.68 | 0.000 |
| Time points of outcome assessment | | | | | | | |
| at discharge 1 0.28(0.02 to 0.53) / / 2.11 0.035 | | | | | | | |
| 3 months after stroke | 9 | 0.59(0.37 to 0.80) | | 91.7 | 0.000 | 5.43 | 0.000 |
| 6 months after stroke | 1 | 0.60(0.21 to 0.99) | | / | / | 3.04 | 0.002 |
| Definition of SHR | | | | | | | |
| FBG/EAG 5 0.55(0.26 to 0.85) 90.4 0.000 3.73 0.000 | | | | | | | |
| FBG/HbA1c 6 0.57(0.32 to 0.81) 87.4 0.000 4.60 0.000 | | | | | | | |
| Raw data representation |  |  | |  |  |  |  |
| Median (IQR) | 4 | 0.69(0.29 to 1.09) | | 96.2 | 0.000 | 3.38 | 0.001 |
| Mean (SD) | 7 | 0.48(0.31 to 0.65) | | 70.5 | 0.002 | 5.56 | 0.000 |

Abbreviations: SMD: standardized mean difference; I^2^: I square; RC: retrospective cohort; PC: prospective cohort; DM: diabetes mellitus; IVT: intravenous thrombolysis; MT: mechanical thrombectomy; SHR: stress hyperglycemia ratio; FBG: fasting blood glucose; HbA1c: Hemoglobin A1c; EAG: estimated average glucose; EAG=(1.59×HbA1c)-2.59; IQR: interquartile range; SD: standard deviation; None: IVT or MT was not performed.

Table S5. Results of meta-regression analysis

| Variables | Coefficients | Standard error | 95% CI | z | p-value |
| --- | --- | --- | --- | --- | --- |
| Study design | 0.134 | 0.220 | -0.385 to 0.653 | 0.61 | 0.560 |
| Country | -0.133 | 0.248 | -0.719 to 0.454 | -0.53 | 0.609 |
| DM | 0.190 | 0.185 | -0.238 to 0.617 | 1.02 | 0.337 |
| Poststroke treatment | 0.166 | 0.255 | -0.438 to 0.770 | 0.65 | 0.537 |
| Time points of outcome assessment | 0.136 | 0.264 | -0.488 to 0.759 | 0.51 | 0.623 |
| Definition of SHR | 0.061 | 0.235 | -0.494 to 0.616 | 0.26 | 0.803 |
| Raw data representation | -0.224 | 0.220 | -0.745 to 0.296 | -1.02 | 0.342 |

Abbreviations: CI: confidence interval; DM: diabetes mellitus; SHR: stress hyperglycemia ratio.

Figure S1. Flowchart of study search and selection


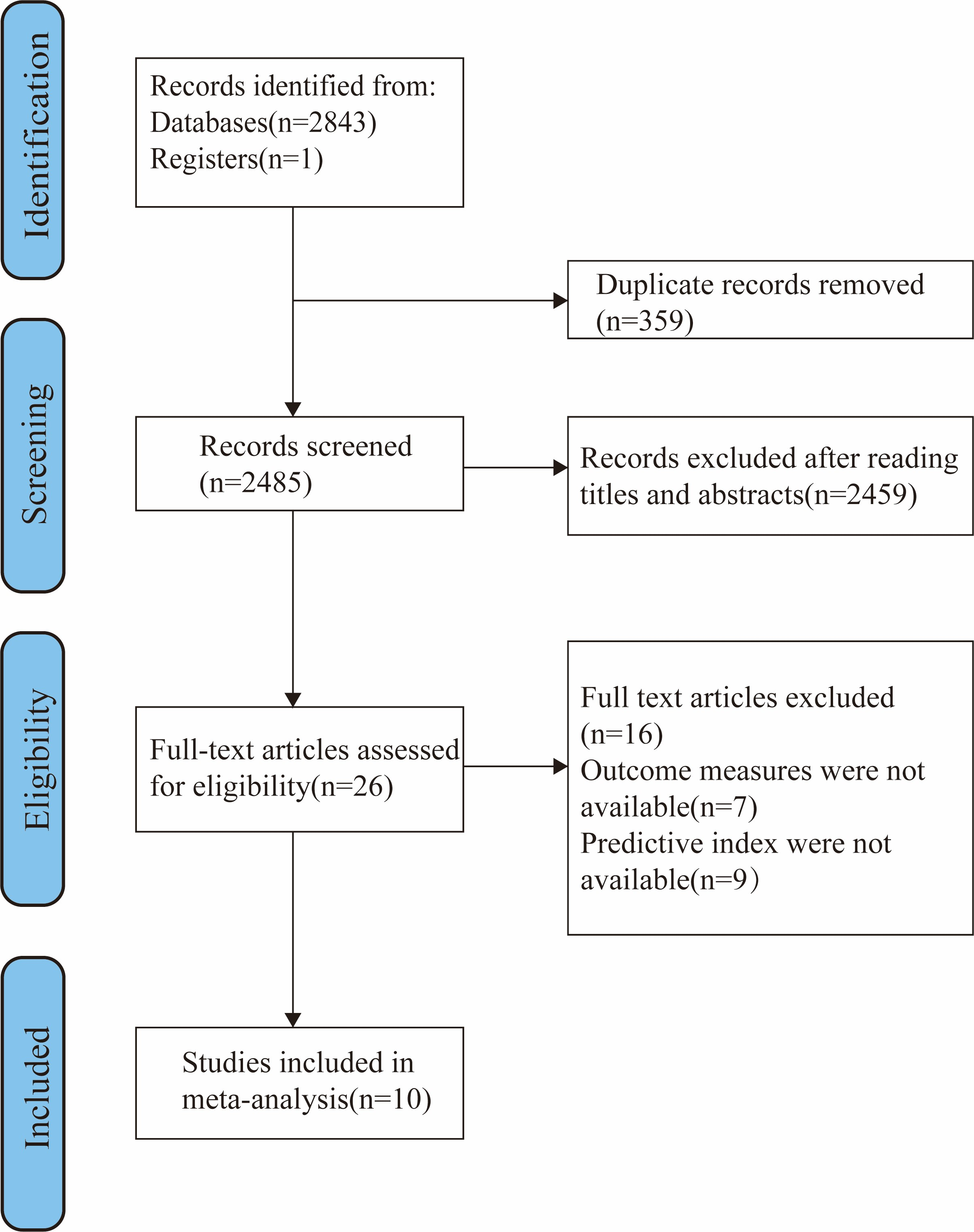


Figure S2. Forest plot of the relationship between SHR and the prognosis of AIS


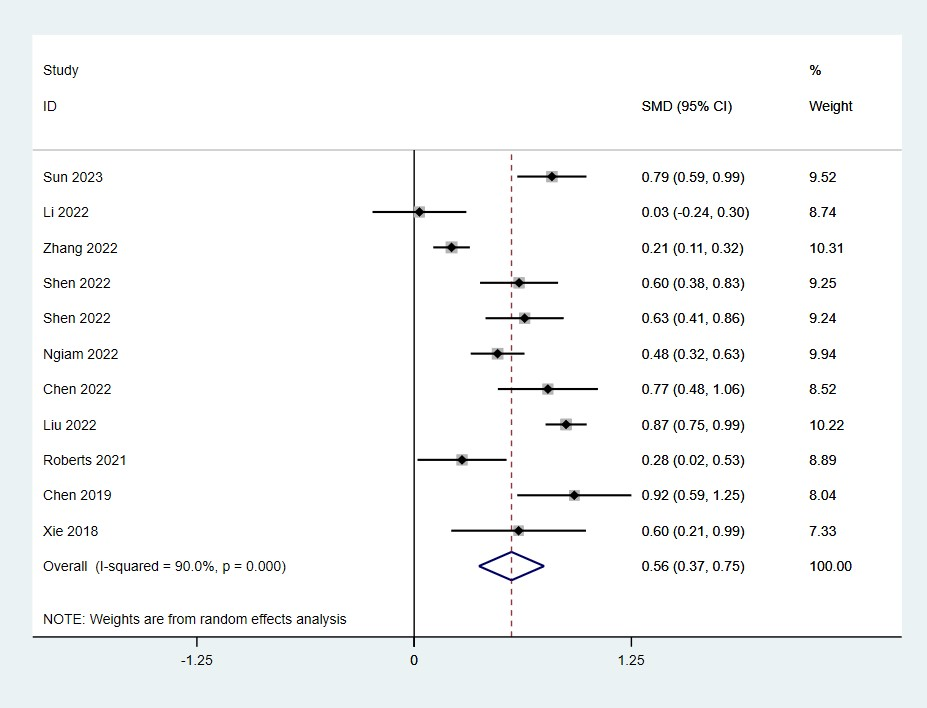


Figure S3. Sensitivity analysis of the meta-analysis


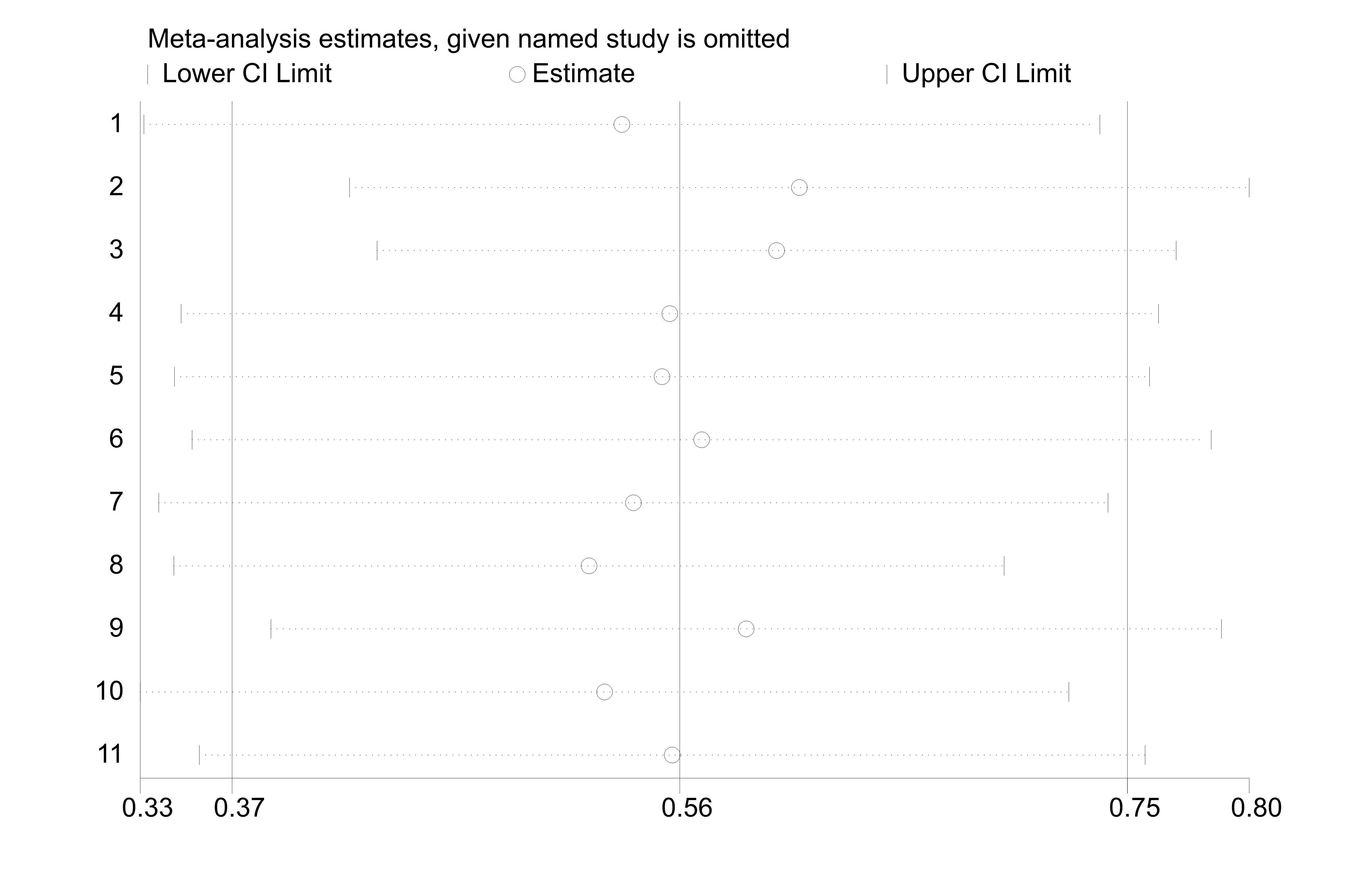


Figure S4. The funnel plot of the meta-analysis


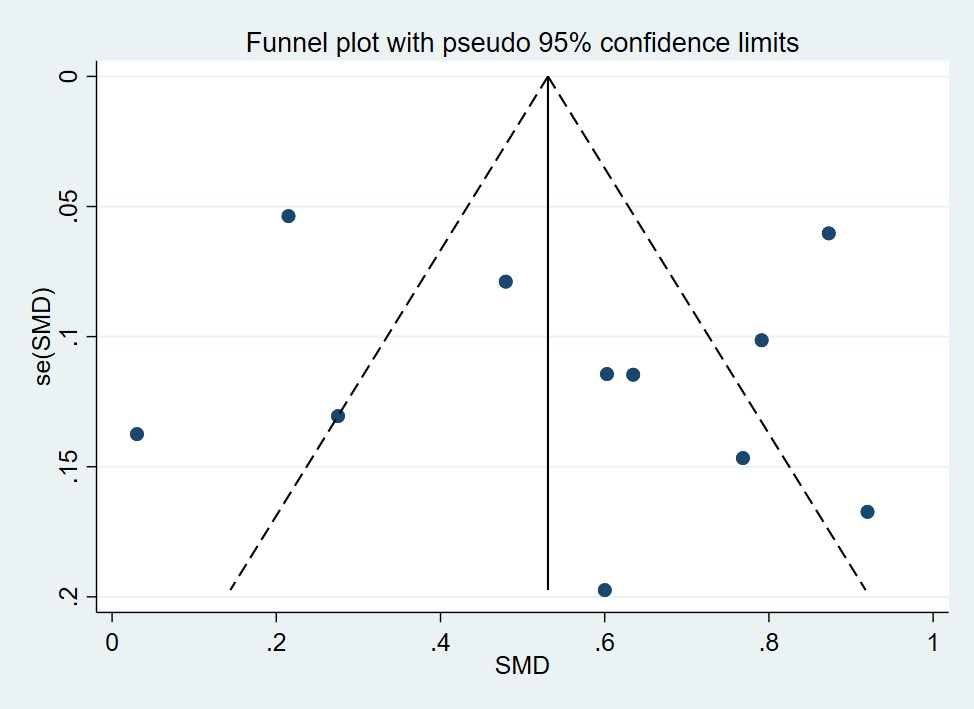


Figure S5. Egger’s test of the meta-analysis


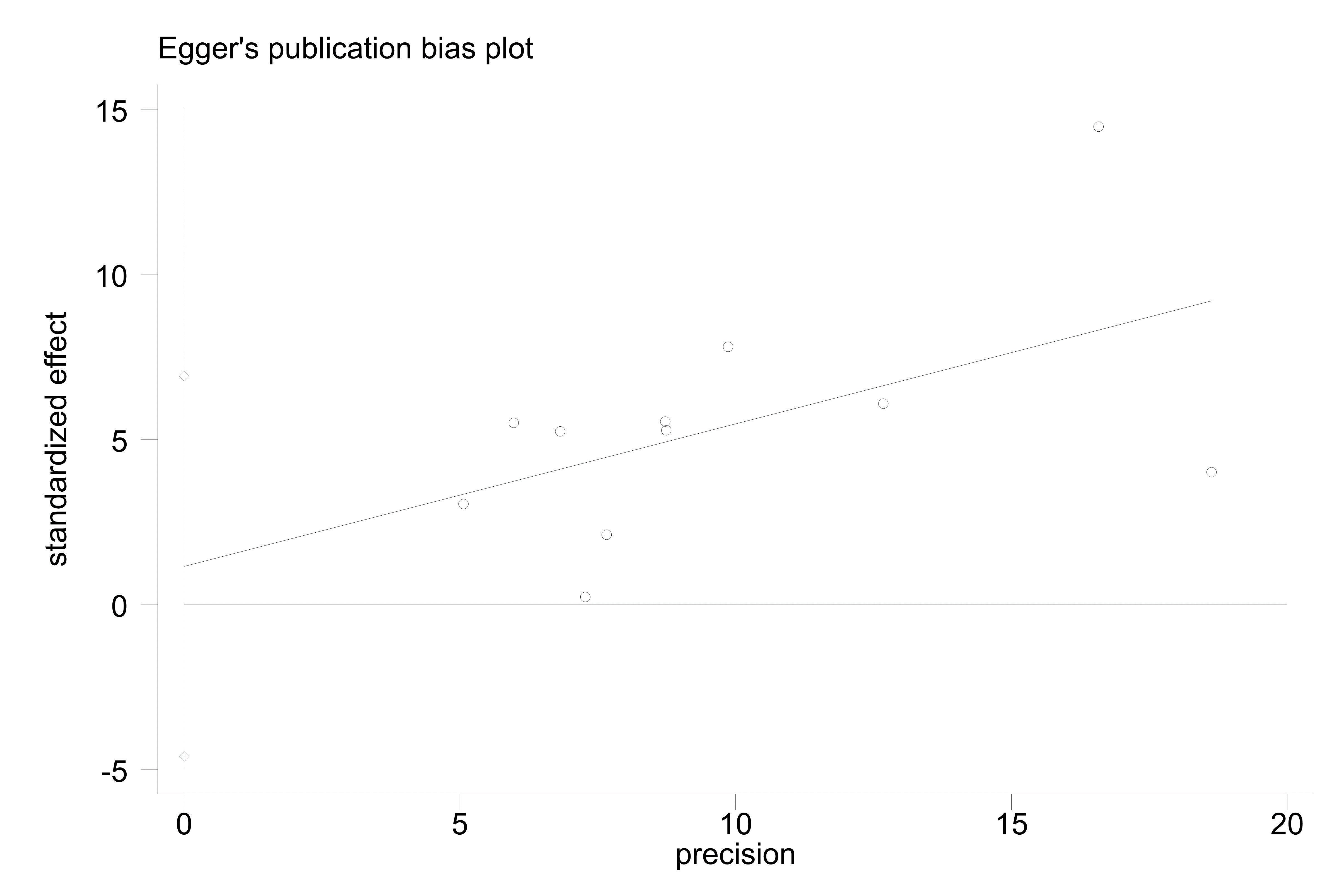

Supplement: Supplementary file 1 — Supplementary Material 1 [file 12883_2023_3519_MOESM1_ESM.docx]
